# Regulation of Phagocyte Migration by Signal Regulatory Protein-Alpha Signaling

**DOI:** 10.1371/journal.pone.0127178

**Published:** 2015-06-09

**Authors:** Julian Alvarez-Zarate, Hanke L. Matlung, Takashi Matozaki, Taco W. Kuijpers, Isabelle Maridonneau-Parini, Timo K. van den Berg

**Affiliations:** 1 Sanquin Research and Landsteiner Laboratory, Academic Medical Center, University of Amsterdam, 1066 CX Amsterdam, The Netherlands; 2 Division of Molecular and Cellular Signaling, Department of Biochemistry and Molecular Biology, Kobe University Graduate School of Medicine, Kobe 650–0017, Japan; 3 Emma Children’s Hospital, Sanquin Research and Landsteiner Laboratory, Academic Medical Center, University of Amsterdam, 1105 AZ, Amsterdam, The Netherlands; 4 CNRS, UMR5089, IPBS (Institut de Pharmacologie et de Biologie Structurale), Toulouse, France; 5 Université de Toulouse, UPS, IPBS, Toulouse, France; Center for Cancer Research, National Cancer Institute, UNITED STATES

## Abstract

Signaling through the inhibitory receptor signal regulatory protein-alpha (SIRPα) controls effector functions in phagocytes. However, there are also indications that interactions between SIRPα and its ligand CD47 are involved in phagocyte transendothelial migration. We have investigated the involvement of SIRPα signaling in phagocyte migration *in vitro* and *in vivo* using mice that lack the SIRPα cytoplasmic tail. During thioglycolate-induced peritonitis in SIRPα mutant mice, both neutrophil and macrophage influx were found to occur, but to be significantly delayed. SIRPα signaling appeared to be essential for an optimal transendothelial migration and chemotaxis, and for the amoeboid type of phagocyte migration in 3-dimensional environments. These findings demonstrate, for the first time, that SIRPα signaling can directly control phagocyte migration, and this may contribute to the impaired inflammatory phenotype that has been observed in the absence of SIRPα signaling.

## Introduction

Phagocytes, including granulocytes and macrophages, play a central role during local inflammation, triggered by infection or by other causes, such as e.g. autoimmunity. Extravasation of phagocytes occurs by a series of highly coordinated events, which consecutively include rolling, firm adhesion, and diapedesis [1]. Intercellular adhesion molecules such as selectins, which mediate rolling, and integrins, which are involved in various other steps of the phagocyte transendothelial migration (TEM) process, play a critical role in this, but exactly how the various events are coordinated is not really understood. Once in the tissue phagocytes use chemotactic cues to migrate through the interstitial tissue matrix to the source of infection or inflammation. Interstitial migration can occur by two mechanistically distinct modes. Amoeboid migration represents a form of crawling, which can only occur in relatively loosely organized extracellular matrices, such as fibrillar collagen. It can be performed by essentially all subpopulations of leukocytes, including granulocytes and macrophages, and it depends on activity of the Rho kinase (ROCK) [2,3,4]. Mesenchymal migration is a mode of migration in dense extracellular matrices that among leukocytes can only be performed by macrophages [2,4]. It involves proteolytic degradation of the matrix and it is known that specialized adhesion structures termed podosomes play a critical role in this type of migration [4,5]. There are many unanswered questions also regarding the signaling events that coordinate the interstitial migration of phagocytes.

Signal regulatory protein alpha (SIRPα), also known as p84, BIT, MFR or SHPS-1, is an inhibitory immunoreceptor selectively expressed in myeloid and neuronal cells [6]. Its extracellular domain contains three extracellular immunoglobulin-(Ig)-like domains through which it binds to the broadly expressed CD47 molecule establishing an interaction that can transduce signals downstream of both receptors [7,8]. The SIRPα intracellular tail encodes four tyrosine residues that form two immunoreceptors tyrosine-based inhibitory motifs (ITIMs). Phosphorylation of these ITIM tyrosines, upon CD47 binding or other stimuli, leads to the recruitment and activation of the cytosolic tyrosine phosphatases Src homology region 2 domain-containing phosphatase-1 and/or 2 (SHP-1 and/or SHP-2). In addition to this, SIRPα has been shown to act as a scaffold for a variety of other signaling and adaptor proteins, such as Src kinase-associated phosphoprotein 2 (SKAP2, Adhesion and Degranulation-promiting protein (ADAP) and Protein tyrosine kinase 2 beta (PYK2) [9]. Collectively these SIRPα-associated signaling molecules regulate a variety of phagocyte effector functions dedicated to homeostasis and host defense, most often in a negative fashion. Intriguingly, studies in chronic autoimmune inflammatory models performed in SIRPα mutant mice have demonstrated that signaling through SIRPα is indispensable for establishing inflammation and the associated clinical symptoms [10,11]. This has mainly been attributed to an essential role for SIRPα signaling in the induction of Th17 responses, which are known to be instrumental in the induction of these different autoimmune inflammatory conditions. In turn this may be linked to a marked decrease in the number of CD11c+ DC in lymphoid tissues of SIRPα-mutant mice [12]. However, it seems possible that SIRPα signaling is also acting in a different fashion to affect inflammation, for instance by directly regulating the functions of phagocytes, including neutrophils and macrophages, that are infiltrating the inflamed site. Interestingly, there is *in vitro* evidence that TEM of neutrophils [13] and monocytes [14] involves interactions between SIRPα and CD47, suggesting a possible direct role of SIRPα in regulating phagocyte extravasation and migration.

In the present study we have addressed the role SIRPα signaling in phagocyte migration *in vivo*. In order to do so we have subjected mice that lack the SIRPα cytoplasmic tail (i.e. SIRPα^Δcyt^ mice) to acute sterile thioglycollate peritonitis. We observed a delayed infiltration of both neutrophils and macrophages into the peritoneum during thioglycolate-induced peritonitis. In addition, we demonstrated that SIRPα signaling is instrumental in TEM. Finally, our *in vitro* experiments demonstrated that the amoeboid but not the mesenchymal mode of interstitial migration of macrophages was impaired in the absence of SIRPα signaling. Collectively, these findings show, for the first time, that SIRPα signaling can directly control phagocyte migration both *in vitro* and *in vivo*, and this contributes to our understanding about the role of SIRPα in immunity and host defense.

## Materials and Methods

### Mice and Peritonitis model

SIRPα^Δcyt^ mice on a C57Bl6/J background, have been described before [15]. Where indicated peritonitis was induced by a single i.p. injection of 1 ml of 4% thioglycollate (Sigma, St Louis, MO, USA) in PBS. At the indicated times after injection the mice were sacrificed and the abdominal cavities were flushed with 5 ml ice cold PBS to harvest the cells. All described animal experiments were approved by the Ethical Committee for Animal Experimentation of the Netherlands Cancer Institute in accordance with Dutch law on animal experimentation. All surgery was performed under isoflurane anesthesia and all efforts were made to minimize suffering.

### Flow cytometry

Extracellular stainings were done to distinguish cell populations from the peritoneum and to determine the levels of integrins on bone marrow derived macrophages (BMDM) and bone marrow derived neutrophils (BMDN). After blocking with anti-CD16/32, cells were stained with Gr1 Ab (clone 1A8), F4/80 Ab, CD3 Ab and B220 Ab to identify populations or with CD11a Ab, CD11b Ab, CD11c Ab, CD18 Ab and CD61 Ab to identify different integrins. All Abs were purchased at eBiosciences (San Diego, CA, USA). Measurements were done in a FACS Calibur flow cytometer (BDbioscience. Bedford, MA, USA).

### Isolation and culture of BMDM and BMDN

Bone marrow cells were cultured 7 days on complete medium with 20 ng/mL recombinant mouse macrophage colony stimulating factor (rmM-CSF) (eBiosciences Bedford, MA, USA) to obtain BMDM. BMDN were isolated from bone marrow with Gr1 magnetic beads (Miltenyi biotec, Bergisch Gladbach Germany) and incubated overnight with human recombinant granulocyte colony stimulating factor (hrG-CSF) (10ng/ml) and mouse recombinant interferon gamma (mrIFN-γ) (50ng/ml) (Peprotech, London, UK).

### BMDM and BMDN transwell migration assay

Chemotaxis of BMDM and BMDN was assessed by means of Fluoroblock inserts (Falcon, Colorado Springs, CO, USA). 6*10^5^ BMDN or BMDM from WT and SIRPα^Δcyt^ mice were labeled with calcein-AM (Molecular Probes, Invitrogen, Carlsbad, CA, USA) and seeded on the upper chamber of 3μm or 8μm pores respectively. C5a (10nM) was used as a chemoattractant. Cell migration was assessed by measuring fluorescence in the lower compartment at 2’ intervals for 1h (BMDN) or 2h (BMDM) with a HTS7000+plate reader (Perkin Elmer, Waltham, MA, USA) at an excitation wavelength of 485 nm and emission wavelength of 535 nm.

### Transendothelial migration assay

Brain endothelial (bEnd5) cells (ATCC, Manassas, VA, USA) were seeded in fibronectin coated μ-slides VI 0.4 (Ibidi, Martinsried, Munchen, Germany) until a confluent monolayer was formed and then stimulated with recombinant mouse tumor necrosis factor alpha (rmTNFα) (10ng/ml) (Biovision, Milpitas, CA, USA) for 3 h. BMDN were then flown over the bEnd5 cells at a rate of 90 dyn/cm², left to adhere for 5’, and flown for another 30’. Transmigrating events were monitored by phase contrast with an Axiovert 200 microscope (Zeiss, Jena, Germany)

### Immunofluorescence microscopy

BMDMs (4 x 10^4^) were seeded on fibronectin-coated Ibidi chambers (μ-Slide VI^0,4^) overnight. Cells were fixed with paraformaldehyde (3.7%; Sigma St, Louis, MO, USA) for 15’, washed, permeabilized with Triton-X100 (0.1%; Sigma, St Louis, MO, USA) for 10’, washed, and stained with anti-vinculin Ab (clone HVin-1, Sigma, St Louis, MO, USA), followed by FITC-conjugated goat anti–mouse (Invitrogen, Carlsbad, CA, USA) and FITC–coupled phalloidin (Molecular Probes, Invitrogen Carlsbad, CA, USA). Slides were visualized on a Zeiss Jena, Germany) observer microscope.

### 3D migration assays

For Matrigel assays, Matrigel (BD Biosciences, Bedford, MA, USA) was poured at 4°C in 24 Transwells inserts (8-μm pores Falcon, Colorado Springs CO, USA) and polymerized as described [16]. Fibrillar collagen I matrices were prepared by mixing bovine collagen (2 mg/ml) (Nutragen, Advance biomatrix San Diego, CA, USA) and rat tail collagen (4 mg/ml) (BD Biosciences, Bedford, MA, USA). The preparation was added to Transwell inserts (8 μm pores, Falcon, Colorado Springs CO, USA) and allowed to polymerize as described [2]. Lower and upper chambers of a transwell system (Nunc, Thermo Fisher, Waltham, MA, USA) were filled in with RPMI 1640 containing 10% FCS and 20 ng/mL rmM-CSF (eBiosciences, Bedford, MA, USA) or 1% FCS and 20 ng/mL rmM-CSF, respectively. After serum starvation for 4 h with RPMI containing 1% FCS BMDMs (2 x 10^4^) cells were seeded in the upper chamber and allowed to migrate. After 48 h z-series images were acquired at 30-μm intervals with a Zeiss (Jena, Germany) observer microscope. The % of migrating cells was determined from z-stack images of the matrix and normalized to the total number of cells in the field of view. Where indicated, the Rho-associated protein kinase (ROCK) inhibitor Y27632 (10 μM) (VWR international, Randor, Pensilvania, USA) was added to the upper and the lower chambers.

### Gelatin degradation assay

Coverslips were coated with 0.2 mg/ml FITC coupled-gelatin (Molecular Probes, Invitrogen, Carlsbad, CA, USA) as previously described [16]. BMDMs (1.5 x 10^5^) were cultured for 16h on gelatin-FITC, fixed, processed for phalloidin staining, and observed as described above. Quantification was done by measuring the pixels of total cell surface and the pixels of gelatin-FITC degradation area using Adobe Photoshop software (Adobe Systems, San Jose, CA, USA). Areas of 100 cells were quantified for each condition in 3 separate experiments.

### Immunohistochemistry

Tissue samples from anterior abdominal wall omentum from WT and SIRPα^Δcyt^ mice were taken, fixed (formalin, 10% Acetic acid), embedded in paraffin, prepared for immunohistochemistry and stained with Gr1-Ab.

### Statistical analysis

All data are presented as mean plus standard error of the mean (SEM). Statistical analyses were performed by unpaired Student t-test using Prism software (GraphPad, version 5.01). P values lower than 0.05 were considered significant.

## Results and Discussion

### SIRPα signaling supports phagocyte recruitment during thioglycolate-induced peritonitis

**Fig 1:**
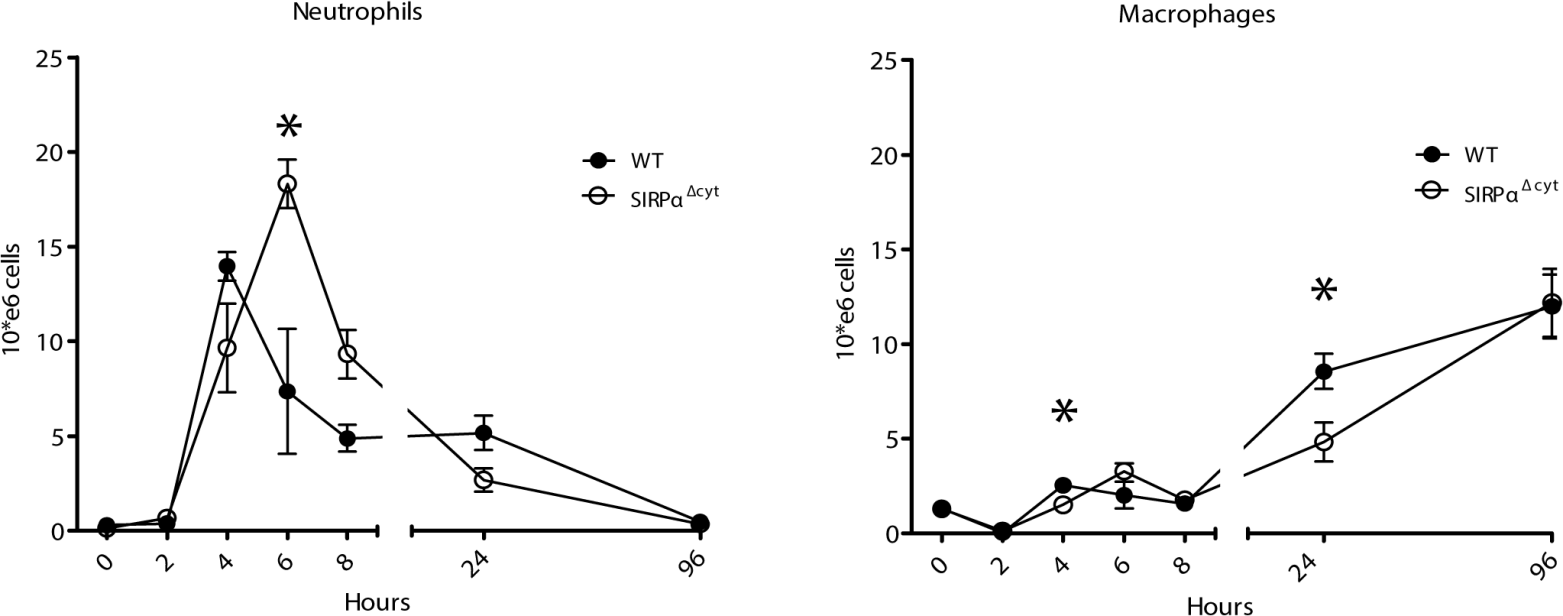
Delayed recruitment of phagocytes to the peritoneal cavity in SIRPα^Δcyt^ mice. After i.p. injection of thioglycolate into WT and SIRPα^**Δcyt**^ mice, neutrophil and macrophage influx were determined in peritoneal lavages at the indicated time points. Total leukocytes were counted and cell populations were discriminated by FACS. Every time point is representative of at least 3 mice. Asterisk, p≤ 0,05. Note that there is a delay in the migration of both SIRPα^**Δcyt**^ phagocyte populations.

To investigate a role for SIRPα signaling in phagocyte migration *in-vivo* we subjected mice lacking the SIRPα cytoplasmic tail (designated SIRPα^Δcyt^)[15] to an acute thioglycollate-induced sterile peritoneal inflammation. Peritoneal lavages were performed to quantify recruitment and determine the composition of the infiltrated cell population. In line with studies previously reported [17,18] phagocyte immigration in wild type (WT) mice occurs in two consecutive waves, which involves first an early influx of predominantly neutrophils peaking at 4 h after thioglycolate injection, followed by an influx of monocytes (which are subsequently defined as macrophages) (Fig 1). While the overall magnitude of the response did not appear to be substantially altered in SIRPα^Δcyt^ mice, a significant delay in the immigration of both neutrophils as well as macrophages (Fig 1) was observed. For instance, in SIRPα^Δcyt^ mice the peak of neutrophil infiltration occurred after about 6 h instead of 4 h, as found in WT mice. No differences in the kinetics and magnitude of lymphocyte migration were observed (data not shown), consistent with a phagocyte-selective effect. Furthermore, there were no significant differences in the blood cell counts of neutrophils and monocytes before and during the experiment (S1A Fig) that could have contributed to the observed delay in phagocyte recruitment and the expression levels of some of the most important integrins for leukocyte extravasation on neutrophils and peripherial blood mononucleated cells (PBMCs) of WT and SIRPα^Δcyt^ showed no difference (S1B Fig). Of interest, the levels of integrins on neutrophils and mononuclear cells (mainly macrophages) remained similar upon extravasation and infiltration to the peritoneal cavity (S1C Fig).

Because SIRPα is primarily expressed on phagocytes [6], including both neutrophils and macrophages, it seemed most likely that the observed supportive role for SIRPα signaling in phagocyte migration was intrinsic to the migrating cells (see also below). However, extrinsic differences, such as e.g. those in the production of chemokines/cytokines, cannot be completely excluded. In this context our preliminary findings upon analysis of peritoneal lavages demonstrated, that there were no notable differences at the early stages of production (i.e. 2 and 6 h) of some of the relevant soluble mediators, such as monocyte chemotactic protein-1 (MCP-1), TNFα and Interleukin-1 beta (IL1β) (data not shown). This is also in line with our previous observation that macrophages from these SIRPα^Δcyt^ animals do not show any differences in cytokine production [9,19]. The most important site of leukocyte recruitment during peritonitis in wild type mice is the omentum, and in particular the part associated to the pancreas and stomach [20,21]. Immunohistochemical analysis showed that leukocytes were trafficking through the same anatomical areas of the omentum in both SIRPα^Δcyt^ and WT mice (S1D Fig).

Taken together, these findings demonstrate a supportive role for SIRPα signaling in phagocyte migration *in vivo* under acute inflammatory conditions, at least those triggered by thioglycollate intra peritoneal injection,. Furthermore, they are consistent with, but do not provide definitive proof for, a cell-autonomous role for SIRPα signaling in phagocyte migration.

### SIRPα^Δcyt^ phagocytes exhibit impaired transendothelial migration and chemotaxis

**Fig 2:**
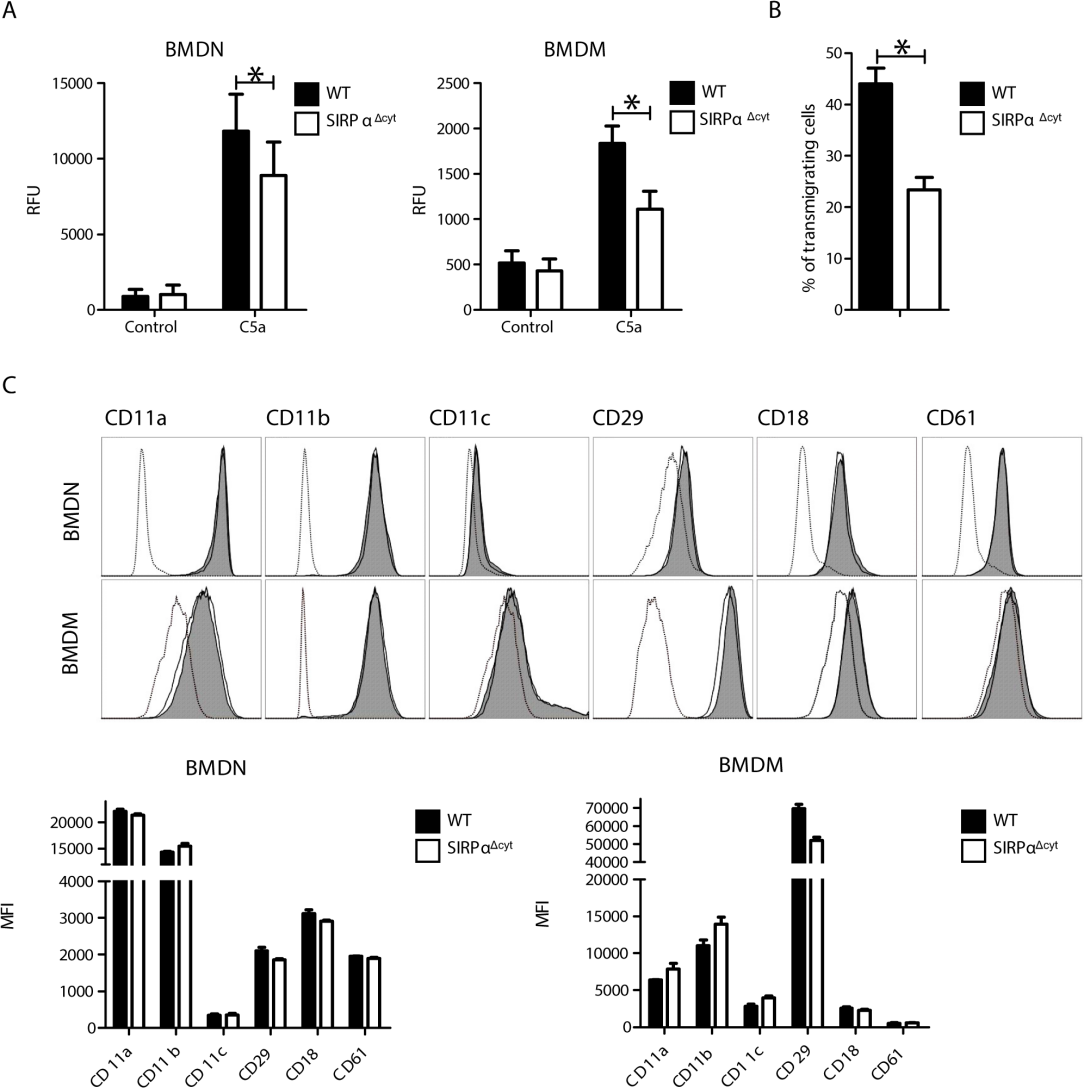
2D transwell chemotaxis and transendothelial migration are impaired in SIRPα^Δcyt^ phagocytes. A) C5a-induced 2D migration in transwell chemotaxis assay is regulated by SIRPα signaling. Data shown represents the difference between the maximum and the minimum fluorescent value reached within 1 h in BMDN and 2 h in BMDM. Values shown represent averages ± SEM of n = 4 independent experiments. Asterisk, p≤0,05. B) Transendothelial migration is deficient in SIRPα^**Δcyt**^ BMDN. WT and SIRPα^**Δcyt**^ BMDN were seeded over a mrTNFα-stimulated monolayer of bEnd5 cells. After 5’ a flow ratio of 0.9dyn/cm^**2**^ was applied and transendothelial migration was monitored by time lapse video microscopy using a phase-contrast lens. Data shown represent means ± SEM of 12 measurements done in 3 independent experiments. Asterisk, p≤0,05. C) BMDN and BMDM from SIRPα^**Δcyt**^ mice have similar levels of integrin expression. Cells were cultured as described in material and methods and stained with specific Abs against the indicated integrins. Gating was based on FCS and SSC. Histograms from representative experiments are shown for BMDN and BMDM. The dotted line represents the isotype control wile the continuous line represents WT (gray filled) or SIRPα^**Δcyt**^ (no filling) stainings.

The migration of leukocytes from the circulation into the peritoneal cavity involves a number of well-defined steps, including extravasation at the postcapillary vessels of the omentum, and the subsequent trafficking through the interstitial tissue towards the peritoneal cavity. In order to study the different aspects of phagocyte migration that are relevant in this context we employed a combination of *in vitro* migration methods, in which cells from wild type and SIRPα^Δcyt^ mice were compared. First, we studied the capacity of neutrophils and macrophages to perform chemotaxis in a standard transwell assay using complement component 5a (C5a) as the chemoattractant. We observed that both SIRPα^Δcyt^ neutrophils and macrophages transmigrated significantly and consistently less as compared to wild type cells (Fig 2A). To investigate whether SIRPα signaling also affected phagocyte transmigration in a more physiological context we employed a transendothelial migration assay in which neutrophils migrate across a monolayer of activated mouse bEnd5 endothelial cells. Due to the difficulty in the isolation of sufficient numbers of mouse monocytes from blood this assay could only be performed with neutrophils. Clearly, SIRPα^Δcyt^ neutrophils displayed considerably less transendothelial migration (Fig 2B, but overall adhesiveness and motility did not appear to be substantially altered (S1 Video). Given the critical importance for integrins in the phagocyte transendothelial migration process [1,22], and the reported role of SIRPα signaling in integrin-mediated cytoskeletal arrangement [9], we investigated whether the observed effects could potentially be due to an altered expression of integrins. As can be seen in Fig 2C there were no significant differences in the levels of expression of many of the relevant integrins. It should be noted that these findings are also consistent with previous observations [9] that demonstrated that SIRPα^Δcyt^ macrophages do not display differences in the relevant β_1,_, β_2_, and β_3_ integrins. Collectively, these results demonstrate that SIRPα signaling in phagocytes contributes to chemotaxis and transendothelial migration. Clearly, this could, at least in part, provide an explanation for the delayed phagocyte influx into the peritoneal cavity described above.

### SIRPα signaling regulates amoeboid but not mesenchymal migration

**Fig 3:**
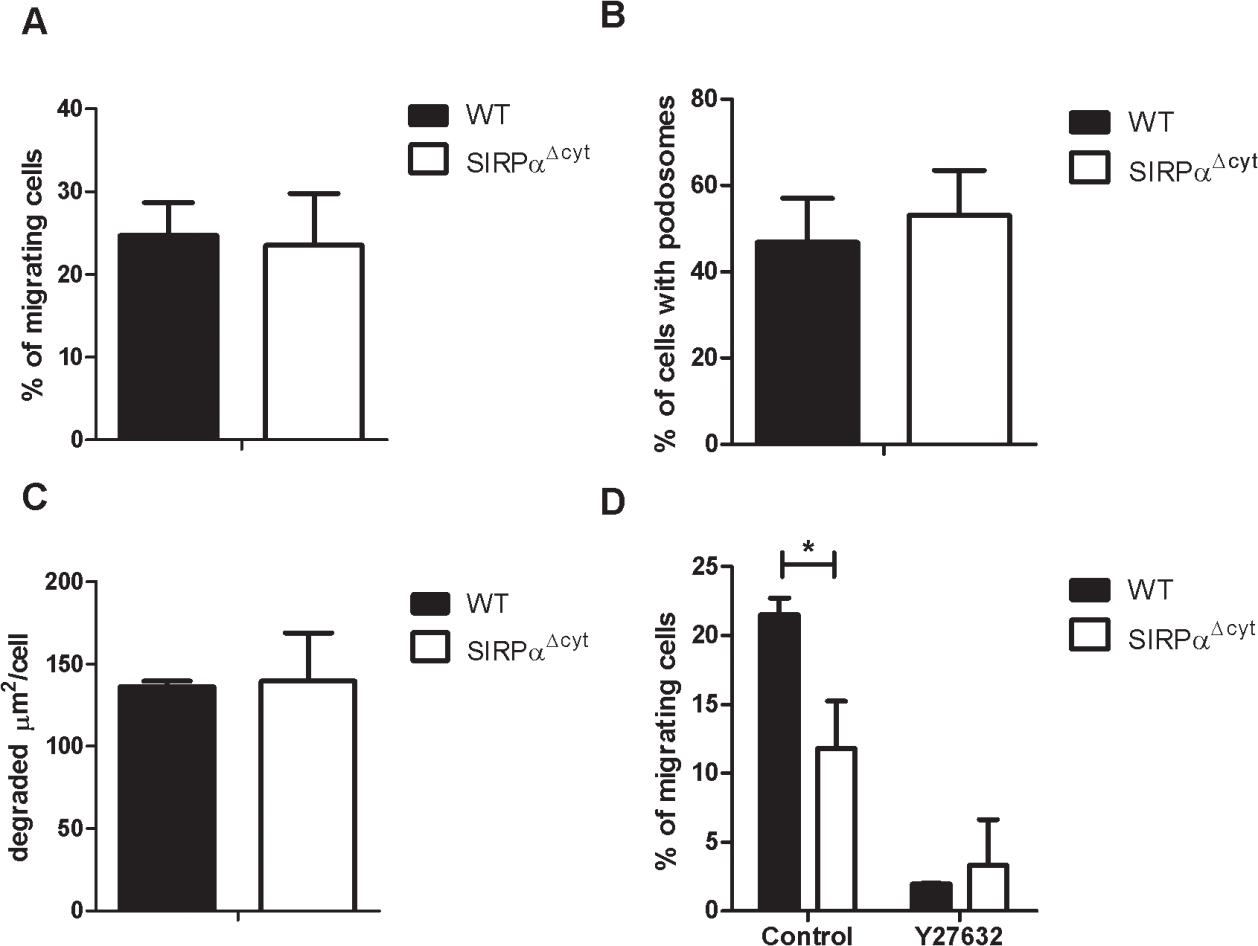
SIRPα signaling in macrophage 3D migration and matrix degradation. A) SIRPα signaling in macrophages does not regulate mesenchymal migration. BMDM were seeded on Matrigel and allowed to migrate for 48 h. Values are averages ± SEM from 3 independent experiments each performed in triplicate. WT cells: black bars, SIRPα^**Δcyt**^: white bars. B) Formation of podosomes in BMDM of SIRPα^**Δcyt**^ mice. BMDM were seeded in fibronectin-coated inserts and were serum starved overnight. Then samples were fixed, permeabilized and stained for F-actin, vinculin and nuclear DNA. Values are averages ± SEM from 3 independent experiments each performed in triplicate. C) SIRPα signaling in macrophage matrix degradation. BMDM were plated on gelatin-FITC coated coverslips and cultured overnight. The samples were then fixed, permeabilized and stained for F-actin and nuclear DNA. The area of degraded gelatin-FITC was quantified and normalized to the area covered by the cells (determined by cortical F-actin staining). Values are averages ± SEM from 3 independent experiments. D) Amoeboid macrophage migration is regulated by SIRPα signaling. Cells were seeded in fibrillar collagen and allowed to migrate for 48 h. To validate the amoeboid mode of migration the ROCK inhibitor Y27632 was used at a concentration of 10μM.Values are averages ± SEM from 3 independent experiments each performed in triplicate. Asterisk, p≤ 0,05.

In addition to the above findings, which are primarily relevant in the context of transendothelial migration, we also wanted to explore a potential role of SIRPα signaling in the subsequent 3D migration of phagocytes in interstitial tissues. Basically, there are two modes of macrophage migration through 3D matrices, designated amoeboid and mesenchymal migration [2,4,23]. Neutrophils exclusively migrate in the amoeboid mode, whereas macrophages can perform either amoeboid or mesenchymal migration depending on the matrix substrate offered [2,4]. To study potential differences between SIRPα^**Δ**cyt^ and WT cells, amoeboid and mesenchymal migration of macrophages was studied in *in vitro* assays [4] Mesenchymal migration involves the formation of specialized adhesion structures, known as podosomes, which are instrumental in proteolytic degradation of the matrix, a process that can be visualized by plating cells on FITC-labelled gelatin [24]. First, we tested the role of SIRPα signaling in mesenchymal migration as assayed by the percentage of macrophages migrating into 3D matrigel gels within 48 h. As can be seen in Fig 3A, no differences between SIRPα^**Δ**cyt^ and WT macrophages were observed. In line with this, podosome formation (Fig 3B) and function, as assayed by gelatin degradation (Fig 3C and S2 Fig), were unaffected by SIRPα mutation, collectively suggesting that mesenchymal migration is normal in SIRPα^**Δ**cyt^ macrophages. In contrary, amoeboid migration in fibrillar collagen I was reduced by more than 50% (Fig 3D). Parallel experiments with the ROCK inhibitor Y27632 provided further confirmation that both WT as well as SIRPα^**Δ**cyt^ macrophages employed this mode of migration. These findings indicate that SIRPα signaling is selectively regulating macrophage amoeboid migration, but not mesenchymal migration.

To conclude, our findings provide direct evidence, for the first time, that SIRPα signaling plays a direct supportive role in phagocyte migration *in vitro* and *in vivo*. In addition to the available data demonstrating that TEM of neutrophils and monocytes requires interactions between phagocyte SIRPα and CD47 on endothelial cells [13,14], our detailed *in vitro* analysis of the different migration properties of SIRPα^**Δ**cyt^ phagocytes provided here, indicates that TEM includes at least one step that is supported by SIRPα signaling, and this could obviously contribute to the delayed phagocyte migration we observed *in vivo*. Moreover, it could be that the reduced interstitial and in particular the amoeboid-type of macrophage migration through tissues found in SIRPα^**Δ**cyt^ mice may also have contributed to this. Intravital microscopic analysis of phagocyte migration would clearly be necessary to obtain more insight with respect to the relative contributions of these effects.

Exactly how the lack of the SIRPα signaling results in these migration deficiencies is not clear yet. Apart from being an inhibitory immunoreceptor that recruits and activates the tyrosine phosphatases SHP-1 and SHP-2, SIRPα has also been shown to be a scaffold protein binding to several intracellular mediators that may transduce signals [25]. While effects of SIRPα signaling on migration in murine embryonic fibroblasts were found to be mediated through SHP-2 [15] other players, such as SKAP2 and ADAP, have recently been implicated in regulating cytoskeletal changes downstream of integrins and SIRPα in macrophages [9]. In particular, it was shown that integrin-induced actin reorganization was impaired in SIRPα^**Δ**cyt^- or SKAP2- deficient murine macrophages, and a critical role for SKAP2-SIRPα interactions and signaling in this context was also implicated. While amoeboid migration, at least in dendritic cells, appears to be largely integrin independent [26] this does of course not exclude a potential role for SIRPα signaling in macrophage amoeboid migration. Maybe even more importantly, integrins and their downsteam signaling and cytoskeletal remodeling are well known to play a prominent role in phagocyte TEM and SKAP2-SIRPα-dependent signals may well be instrumental in this. Nevertheless, further studies will be required to understand the precise role(s) of SIRPα signaling in phagocyte migration.

Finally, the role of interactions between SIRPα on phagocytes and CD47 on endothelial cells during TEM needs to be clarified although a number of *in vitro* studies suggested that such interactions were required during TEM [13,14]. Furthermore, *in vivo* studies in CD47-deficient mice suggested a role for CD47 in *S*. *aureus*-induced peritonitis consistent with the involvement of CD47-SIRPα interactions in phagocyte extravasation. However, more recent studies did not confirm this when normal infiltration of polymorphonuclear cells (PMNs) was observed in CD47^-/-^ mice upon peritoneal insult [27]. In the context of *in vitro* migration into fibrillar collagen I, SIRPα on the migrating macrophages can not be ligated by extracellular CD47 essentially, suggesting regulation of interstitial amoeboid migration by either *cis* CD47-SIRPα interactions or by ligand-independent SIRPα signaling.

## Supporting Information

S1 Fig Part A) Blood counts of neutrophils and monocytes do not differ between WT and SIRPα^Δcyt^. After i.p. injection of thioglycolate into WT and SIRPα^Δcyt^ mice neutrophil and monocytes blood counts were determined at the indicated time points. Total leukocytes were counted and cell populations were discriminated by FACS. Every time point is representative of at least 3 mice. Part B) Neutrophils and PBMC from SIRPα^Δcyt^ mice have similar levels of integrin expression than those of WT. Blood samples were taken at 6h after thioglycollate injection. Blood was lysed and stained for integrins. Neutrophils and PBMC were discriminated based on FSC and SSC. Graphs represents averages ± SEM of at least 3 mice per group. Part C) Neutrophils and mononuclear cells (MC) from the peritoneal cavity of SIRPα^Δcyt^ mice have similar levels of integrin expression than those of WT. Peritoneal samples were taken at 6h after thioglycollate injection and stained for integrins. Neutrophils and MC were discriminated based on FSC and SSC. Graphs represents averages ± SEM of at least 3 mice per group. Part D) Neutrophils from SIRPα^Δcyt^ and WT mice extravasate through the stomach and pancreas- associated omentum. Sections from thioglycollate injected mice were prepared for immunohistochemistry and staining with anti-Ly6G.
(TIF)

S2 Fig Gelatin degradation by WT and SIRPα^Δcyt^ macrophages. BMDM were plated on gelatin-FITC coated coverslips and cultured overnight. After fixation, samples were stained for F-actin (phalloidin, red) and nuclei were stained with DAPI (Blue). Note that the black areas represent the regions of gelatin degradation by BMDM. Left panels show BMDM, middle panels show gelatin-FITC degradation and right panels are merged images.
(TIF)

S1 Video Transendothelial migration of BMDN is regulated by SIRPα signaling. BMDN were flown over a monolayer of endothelial cells for 5 min, allowed to rest on the endothelial cells for other 5 min and then subjected to flow conditions for 20 min. Transmigrating events were recognize as phase-contrast negative BMDN.
(MPG)

## References

[1] Ley K., Laudanna C., Cybulsky M.I. and Nourshargh S. (2007) Getting to the ite of inflammation: the leukocyte adhesion cascade updated. Nat. Rev. Immunol., 7, 678–689. 17717539 10.1038/nri2156

[2] Cougoule C., Van G.E., Le C., V, Lafouresse F., Dupre L., Mehraj V., et al (2012) Blood leukocytes and macrophages of various phenotypes have distinct abilities to form podosomes and to migrate in 3D environments. Eur. J. Cell Biol., 91, 938–949. 10.1016/j.ejcb.2012.07.002 22999511

[3] Lammermann T. and Sixt M. (2009) Mechanical modes of 'amoeboid' cell migration. Curr. Opin. Cell Biol., 21, 636–644. 10.1016/j.ceb.2009.05.003 19523798

[4] Van G.E., Poincloux R., Gauffre F., Maridonneau-Parini I. and Le C., V (2010) Matrix architecture dictates three-dimensional migration modes of human macrophages: differential involvement of proteases and podosome-like structures. J. Immunol., 184, 1049–1061. 10.4049/jimmunol.0902223 20018633

[5] Van G.E., Guiet R., Balor S., Charriere G.M., Poincloux R., Labrousse A., et al (2011) Macrophage podosomes go 3D. Eur. J. Cell Biol., 90, 224–236. 10.1016/j.ejcb.2010.07.011 20801545

[6] Adams S., van der Laan L.J., Vernon-Wilson E., Renardel de L.C., Dopp E.A., Dijkstra C.D., et al (1998) Signal-regulatory protein is selectively expressed by myeloid and neuronal cells. J. Immunol., 161, 1853–1859. 9712053

[7] Isenberg J.S., Frazier W.A., Krishna M.C., Wink D.A. and Roberts D.D. (2008) Enhancing cardiovascular dynamics by inhibition of thrombospondin-1/CD47 signaling. Curr. Drug Targets., 9, 833–841. 18855617 10.2174/138945008785909338 PMC2575641

[8] Matozaki T., Murata Y., Okazawa H. and Ohnishi H. (2009) Functions and molecular mechanisms of the CD47-SIRPalpha signalling pathway. Trends Cell Biol., 19, 72–80. 10.1016/j.tcb.2008.12.001 19144521

[9] Alenghat,F.J., Baca,Q.J., Rubin,N.T., Pao,L.I., Matozaki,T., Lowell,C.A., et al. (2012) Macrophages require Skap2 and Sirpalpha for integrin-stimulated cytoskeletal rearrangement. J. Cell Sci. 10.1242/jcs.111260 PMC3561861 22976304

[10] Okuzawa C., Kaneko Y., Murata Y., Miyake A., Saito Y., Okajo J., et al (2008) Resistance to collagen-induced arthritis in SHPS-1 mutant mice. Biochem. Biophys. Res. Commun., 371, 561–566. 10.1016/j.bbrc.2008.04.124 18455510

[11] Tomizawa T., Kaneko Y., Kaneko Y., Saito Y., Ohnishi H., Okajo J., et al (2007) Resistance to experimental autoimmune encephalomyelitis and impaired T cell priming by dendritic cells in Src homology 2 domain-containing protein tyrosine phosphatase substrate-1 mutant mice. J. Immunol., 179, 869–877. 17617577 10.4049/jimmunol.179.2.869

[12] Saito Y., Iwamura H., Kaneko T., Ohnishi H., Murata Y., Okazawa H., et al (2010) Regulation by SIRPalpha of dendritic cell homeostasis in lymphoid tissues. Blood, 116, 3517–3525. 10.1182/blood-2010-03-277244 20682853

[13] Liu Y., Buhring H.J., Zen K., Burst S.L., Schnell F.J., Williams I.R. et al (2002) Signal regulatory protein (SIRPalpha), a cellular ligand for CD47, regulates neutrophil transmigration. J. Biol. Chem., 277, 10028–10036. 11792697 10.1074/jbc.M109720200

[14] de Vries H.E., Hendriks J.J., Honing H., De Lavalette C.R., van der Pol S.M., Hooijberg E., et al (2002) Signal-regulatory protein alpha-CD47 interactions are required for the transmigration of monocytes across cerebral endothelium. J. Immunol., 168, 5832–5839. 12023387 10.4049/jimmunol.168.11.5832

[15] Inagaki K., Yamao T., Noguchi T., Matozaki T., Fukunaga K., Takada T., et al (2000) SHPS-1 regulates integrin-mediated cytoskeletal reorganization and cell motility. EMBO J., 19, 6721–6731. 11118207 10.1093/emboj/19.24.6721 PMC305898

[16] Cougoule C., Carreno S., Castandet J., Labrousse A., Astarie-Dequeker C., Poincloux R., et al (2005) Activation of the lysosome-associated p61Hck isoform triggers the biogenesis of podosomes. Traffic., 6, 682–694. 15998323 10.1111/j.1600-0854.2005.00307.x

[17] Wu Q., Feng Y., Yang Y., Jingliu, Zhou W., He P., et al (2004) Kinetics of the phenotype and function of murine peritoneal macrophages following acute inflammation. Cell Mol. Immunol., 1, 57–62. 16212922

[18] Call D.R., Nemzek J.A., Ebong S.J., Bolgos G.L., Newcomb D.E. and Remick D.G. (2001) Ratio of local to systemic chemokine concentrations regulates neutrophil recruitment. Am. J. Pathol., 158, 715–721. 11159209 10.1016/S0002-9440(10)64014-X PMC1850325

[19] van Beek E.M., Zarate J.A., van B.R., Schornagel K., Tool A.T., Matozaki T., et al (2012) SIRPalpha controls the activity of the phagocyte NADPH oxidase by restricting the expression of gp91(phox). Cell Rep., 2, 748–755. 10.1016/j.celrep.2012.08.027 23022485

[20] Cranshaw M.L. and Leak L.V. (1990) Milky spots of the omentum: a source of peritoneal cells in the normal and stimulated animal. Arch. Histol. Cytol., 53 Suppl, 165–177. 2252628 10.1679/aohc.53.suppl_165

[21] Shah S., Lowery E., Braun R.K., Martin A., Huang N., Medina M., et al (2012) Cellular basis of tissue regeneration by omentum. PLoS. One., 7, e38368 10.1371/journal.pone.0038368 22701632 PMC3368844

[22] Vestweber D. (2007) Adhesion and signaling molecules controlling the transmigration of leukocytes through endothelium. Immunol. Rev., 218, 178–196. 17624953 10.1111/j.1600-065X.2007.00533.x

[23] Friedl P. (2004) Prespecification and plasticity: shifting mechanisms of cell migration. Curr. Opin. Cell Biol., 16, 14–23. 15037300 10.1016/j.ceb.2003.11.001

[24] Linder S. and Aepfelbacher M. (2003) Podosomes: adhesion hot-spots of invasive cells. Trends Cell Biol., 13, 376–385. 12837608 10.1016/s0962-8924(03)00128-4

[25] Timms J.F., Swanson K.D., Marie-Cardine A., Raab M., Rudd C.E., Schraven B. et al (1999) SHPS-1 is a scaffold for assembling distinct adhesion-regulated multi-protein complexes in macrophages. Curr. Biol., 9, 927–930. 10469599 10.1016/s0960-9822(99)80401-1

[26] Lammermann T., Bader B.L., Monkley S.J., Worbs T., Wedlich-Soldner R., Hirsch K., et al (2008) Rapid leukocyte migration by integrin-independent flowing and squeezing. Nature, 453, 51–55. 10.1038/nature06887 18451854

[27] Bian Z., Guo Y., Luo Y., Tremblay A., Zhang X., Dharma S., et al (2013) CD47 deficiency does not impede polymorphonuclear neutrophil transmigration but attenuates granulopoiesis at the postacute stage of colitis. J. Immunol., 190, 411–417. 10.4049/jimmunol.1201963 23203922 PMC5874804

