# Supplementary figures and images for: Regulation of Phagocyte Migration by Signal Regulatory Protein-Alpha Signaling
Source: PLoS One. 2015 Jun 9;10(6):e0127178. doi: 10.1371/journal.pone.0127178 (PMC4461249; doi:10.1371/journal.pone.0127178)

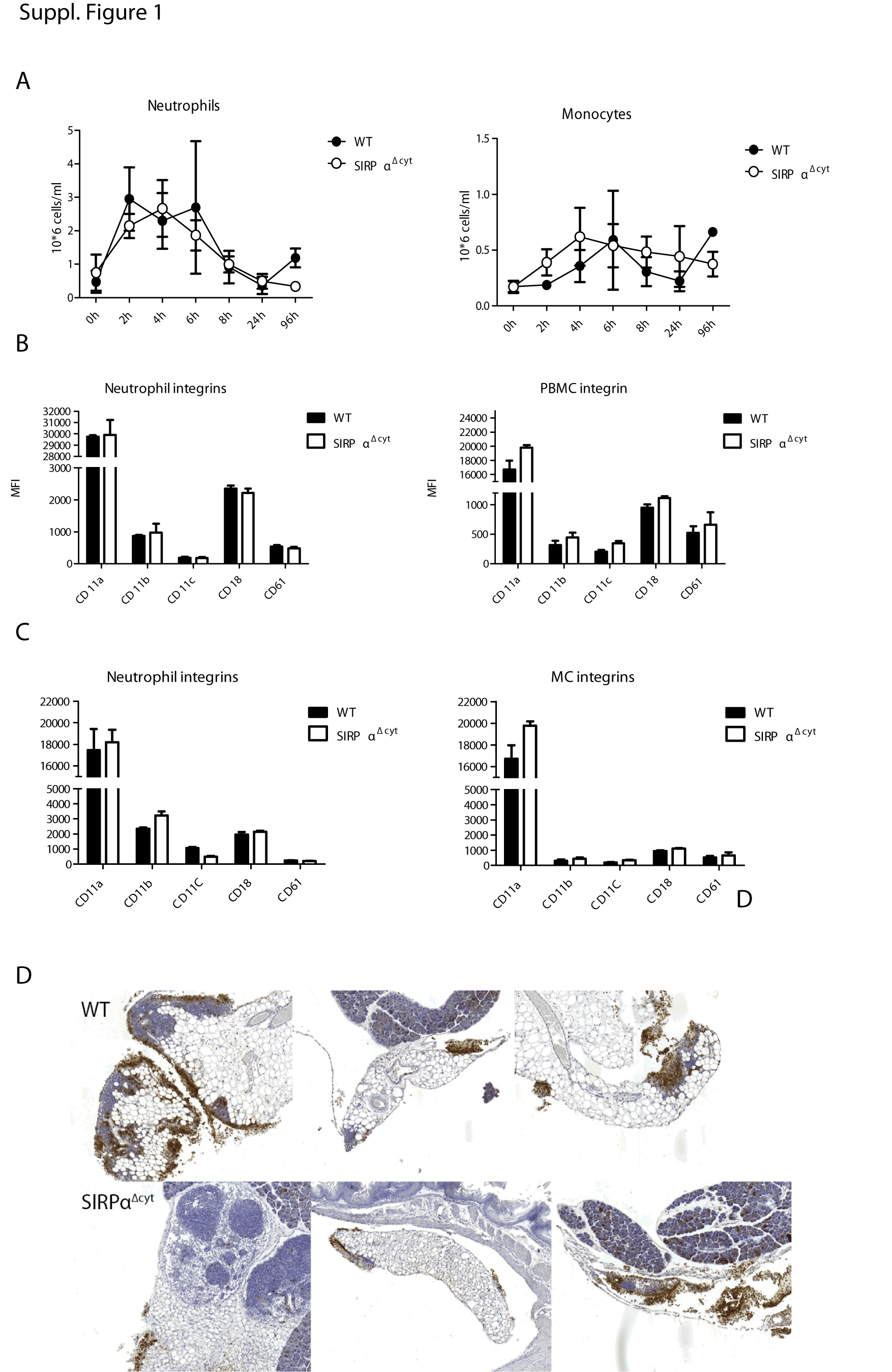

Supplement: S1 Fig — After i.p. injection of thioglycolate into WT and SIRPαΔcyt mice neutrophil and monocytes blood counts were determined at the indicated time points. Total leukocytes were counted and cell populations were discriminated by FACS. Every time point is representative of at least 3 mice. Part B) Neutrophils and PBMC from SIRPαΔcyt mice have similar levels of integrin expression than those of WT. Blood samples were taken at 6h after thioglycollate injection. Blood was lysed and stained for integrins. Neutrophils and PBMC were discriminated based on FSC and SSC. Graphs represents averages ± SEM of at least 3 mice per group. Part C) Neutrophils and mononuclear cells (MC) from the peritoneal cavity of SIRPαΔcyt mice have similar levels of integrin expression than those of WT. Peritoneal samples were taken at 6h after thioglycollate injection and stained for integrins. Neutrophils and MC were discriminated based on FSC and SSC. Graphs represents averages ± SEM of at least 3 mice per group. Part D) Neutrophils from SIRPαΔcyt and WT mice extravasate through the stomach and pancreas- associated omentum. Sections from thioglycollate injected mice were prepared for immunohistochemistry and staining with anti-Ly6G. (TIF) [file pone.0127178.s001.tif]

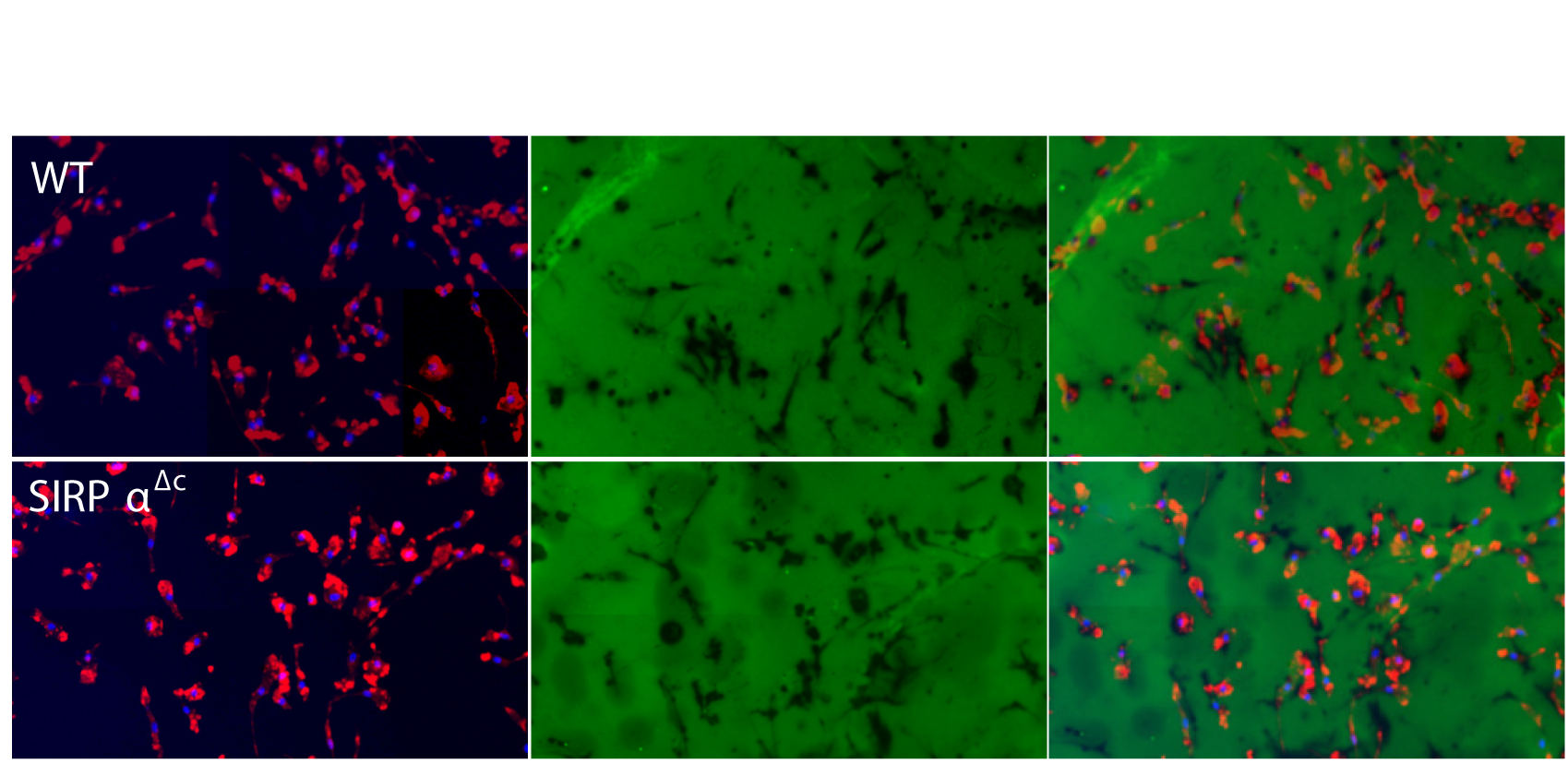

Supplement: S2 Fig — BMDM were plated on gelatin-FITC coated coverslips and cultured overnight. After fixation, samples were stained for F-actin (phalloidin, red) and nuclei were stained with DAPI (Blue). Note that the black areas represent the regions of gelatin degradation by BMDM. Left panels show BMDM, middle panels show gelatin-FITC degradation and right panels are merged images. (TIF) [file pone.0127178.s002.tif]
